# The Effects of Selected Sesquiterpenes from *Myrica rubra* Essential Oil on the Efficacy of Doxorubicin in Sensitive and Resistant Cancer Cell Lines

**DOI:** 10.3390/molecules22061021

**Published:** 2017-06-20

**Authors:** Martin Ambrož, Petra Matoušková, Adam Skarka, Martina Zajdlová, Kateřina Žáková, Lenka Skálová

**Affiliations:** 1 Department of Biochemical Sciences, Faculty of Pharmacy, Charles University, Heyrovského 1203, 50005 Hradec Králové, Czech Republic; ambrozm@faf.cuni.cz (M.A.); matousp7@faf.cuni.cz (P.M.); m.zajdlova@gmail.com (M.Z.); Katerina.zakova@gmail.com (K.Ž.); 2 Department of Chemistry, Faculty of Science, University of Hradec Králové, Hradecká 1285, 50003 Hradec Králové, Czech Republic; adam.skarka@uhk.cz

**Keywords:** terpenes, drug resistance, Adriamycin, drug combinations, ABCB1 transporter

## Abstract

β-caryophyllene oxide (CAO), α-humulene (HUM), trans-nerolidol (NER) and valencene (VAL) are constituents of the essential oil of *Myrica rubra* (MEO), which has significant antiproliferative effect in various cancer cell lines. In the present study, we compared the antiproliferative effect of these sesquiterpenes alone and in combination with the cytostatic drug doxorubicin (DOX) in cancer cell lines with different sensitivity to DOX. Two ovarian cancer cell lines (sensitive A2780 and partly resistant SKOV3) and two lymphoblast cancer cell lines (sensitive CCRF/CEM and completely resistant CEM/ADR) were used. The observed effects varied among sesquiterpenes and also differed in individual cell lines, with only VAL being effective in all the cell lines. A strong synergism of DOX with NER was found in the A2780 cells, while DOX acted synergistically with HUM and CAO in the SKOV3 cells. In the CCRF/CEM cells, a synergism of DOX with CAO and NER was observed. In resistant CEM/ADR cells, sesquiterpenes did not increase DOX efficacy, although they significantly increased accumulation of DOX (up to 10-times) and rhodamine-123 (substrate of efflux transporter ABCB1) within cancer cells. In conclusion, the tested sesquiterpenes were able to improve DOX efficacy in the sensitive and partly resistant cancer cells, but not in cells completely resistant to DOX.

## 1. Introduction

Sesquiterpenes, defined as 15-carbon compounds formed from 3 isoprenoid units, represent an extremely diverse, heterogeneous and large group of natural compounds. Sesquiterpenes are secondary metabolites, formed mainly in plants, fungi, bacteria and marine invertebrates [1,2,3]. Together with monoterpenes, sesquiterpenes represent the major components of plant essential oils, widely used in folk medicines, health-supporting preparations and cosmetics. Many sesquiterpenes possess a broad spectrum of interesting biological activities including anti-cancer ones [4]. They have shown promising effects in various cancer cell lines, where they interfered with the cell cycle and apoptosis as well as increased the production of reactive oxygen species (ROS) and affected cell migration and adhesion [5,6,7].

In addition, several sesquiterpenes were able to enhance the efficacy of classical anticancer drugs in vitro. For example, a combination of the α-methylene sesquiterpene lacton eremophila-1(10)-11(13)-dien-12, 8β-olide with cisplatin or paclitaxel increased the effect of these cytostatics in the ovarian cell lines SKOV3, OVCAR-3, JC and JCpl [8]. Apogossypoyl enhanced the effect of the synthetic tetracycline minocyclin in the pancreatic cancer cell lines MIA PaCa-2, PANC-1, BxPC-3, AsPC-1 and HPNE through inhibition of proteins from the BCL-2 family [9]. Oxaliplatin in combination with β-elemene were more potent in the inhibition of the proliferation of hepatocellular carcinoma cells than oxaliplatin alone. This was caused by increased platinum accumulation and platinum-DNA adducts formation [10]. Our previous studies revealed the synergistic effect of four sesquiterpenes from *Myrica rubra* essential oil, α-humulene (HUM), caryophyllene oxide (CAO), trans-nerolidol (NER) and valencene (VAL), with doxorubicin (DOX) in the CaCo-2 cancer cell line [11]. DOX is a frequently used antineoplastic agent, but aside from its serious side effects (mainly cardiotoxicity), the resistance of many cancer cells decreases the success of its use. Drug resistance is common complication of cancer therapy. Up to 20% of pediatric patients with acute lymphoblastic leukemia have a relapse associated with drug-resistance [12,13]. There is a broad spectrum of mechanisms of drug-resistance e.g., increased drug efflux, alteration of therapy target, increase of drug inactivation, damage repair or inhibition of cell death signaling etc. [14]. Resistance to DOX is mainly ascribed to an increase in ATP-binding cassette (ABC) transporters expression, in particular ABCB1 and ABCC1 [15,16].

The present study was designed to test and compare the effects of sesquiterpenes HUM, CAO, NER and VAL on cell proliferation and DOX efficacy in ovarian and lymphoblast cancer cells with different sensitivity to DOX. Moreover, the ability of these sesquiterpenes to enhance DOX concentration within cells and to inhibit efflux transporters was also tested. Two ovarian cancer cell lines—A2780 (sensitive to DOX) and SKOV3 (partly resistant to DOX)—and two lymphoblast cancer cell lines—CCRF/CEM (DOX-sensitive) and CEM/ADR (DOX-resistant)—were used for this purpose. The amount of the ABCB1 efflux transporter in these cell lines was quantified and compared.

## 2. Results and Discussion

The frequent occurrence of resistance of cancer cells to DOX underscores the necessity of determining compounds which are able to suppress this resistance. These compounds might be detected among sesquiterpenes, as these substances are able to enhance drug uptake via increased membrane permeability. In addition, some sesquiterpenes themselves have been shown to have anti-proliferative effects in cancer cells as well as to act synergistically with classic cytostatics [4]. In a previous study, we showed the ability of *Myrica rubra* essential oil and its sesquiterpene components HUM, CAO, NER and VAL to inhibit proliferation of colon cancer cell lines as well as to increase the efficacy of DOX treatment; this is probably via increased production of reactive oxygen species (ROS) and DOX accumulation in these cells. On the other hand, no cytotoxic effect or amelioration of DOX toxicity was observed in rat hepatocytes as a model of non-cancerous cells [11,17,18]. In the present study, we tested the effect of HUM, CAO, NER, VAL alone and in their combinations with DOX on cell proliferation in two ovarian and two lymphoblast cell lines with different sensitivity to DOX. The A2780 ovarian cancer cell line is sensitive to DOX and other cytostatics, while the SKOV3 ovarian cancer cell line exhibits lower sensitivity to several cytotoxic drugs like cisplatin and DOX. CCRF/CEM is a lymphoblast cancer cell line isolated from peripheral blood taken from a child with acute leukemia. CEM/ADR is a resistant variant of the CCRF/CEM cell line, the resistance of which was developed by repeated incubation of these cells with gradually increased DOX (Adriamycin, ADR) concentration in a culture medium.

### 2.1. Effect of Sesquiterpenes on Cancer Cell Proliferation

**Figure 1:**
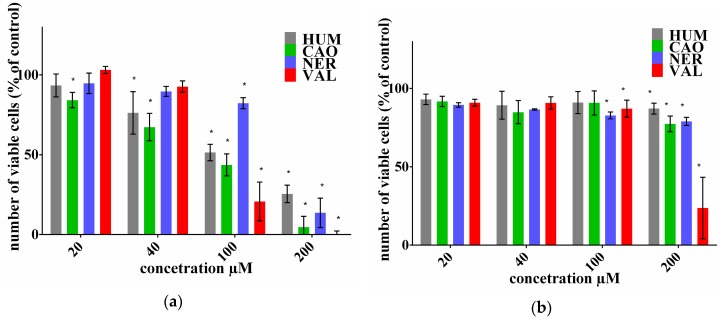
Effect of sesquiterpenes on the proliferation of ovarian cell lines A2780 (**a**) and SKOV3 (**b**). The number of viable cells was assayed using NRU. Data presented as a percentage share of controls (=100%) represent the mean ± S.D. calculated from 3 independent measurements (6 parallels in each). The asterisk indicates a significant difference from the control cells (*p* < 0.05).

**Figure 2:**
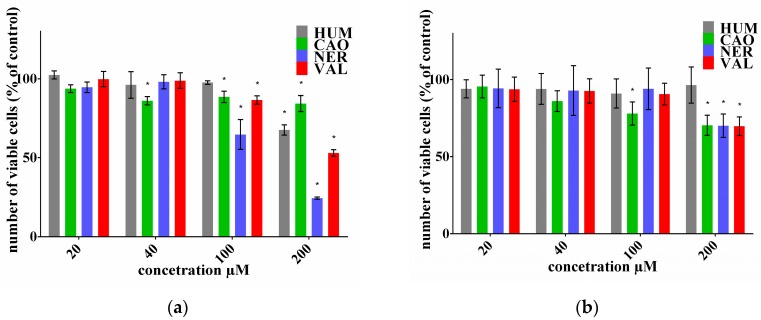
Effect of sesquiterpenes on proliferation of lymphoblast cell lines CCRF/CEM (**a**) and CEM/ADR (**b**). The number of viable cells was assayed using AB. Data presented as a percentage share of controls (=100%) represent the mean ± S.D. calculated from 3 independent measurements (6 parallels in each). The asterisk indicates a significant difference from the control cells (*p* < 0.05).

The effect of selected sesquiterpenes (HUM, CAO, NER and VAL) on the number of viable cells after 72-h treatment was tested using a neutral red uptake (NRU) or Alamar Blue (AB) assay in ovarian cell lines and lymphoblast cell lines, respectively. The results are demonstrated in Figure 1 and Figure 2. The IC_50_ values for individual sesquiterpenes and DOX alone were calculated as presented in Table 1. Proliferation of ovarian cancer cell line A2780 was inhibited by all the tested sesquiterpenes in a dose dependent manner, with CAO being the most effective. Our results correspond with previously reported effects of various sesquiterpenes from *Laurus nobilis* L. in this cancer cell line [19]. On the other hand, ovarian cancer cell line SKOV3, with a decreased sensitivity to DOX, was also much less sensitive to individual sesquiterpenes. Only VAL in the highest concentration was distinctly effective. Lymphoblast cancer cells CCRF/CEM proliferation was inhibited by all tested sesquiterpenes, but only in higher concentrations. NER was the most effective, while CAO had the lowest effect. Cell line CEM/ADR was completely resistant to DOX and it also exhibited low sensitivity to the tested sesquiterpenes. On the other hand, essential oils from *Juniperus*, *Pinus* and *Cedrus* were able to inhibit cell proliferation in both CCRF/CEM and CEM/ADR lines [20].

### 2.2. Effect of Sesquiterpenes in Combinations with DOX on Cancer Cell Proliferation

**Figure 3:**
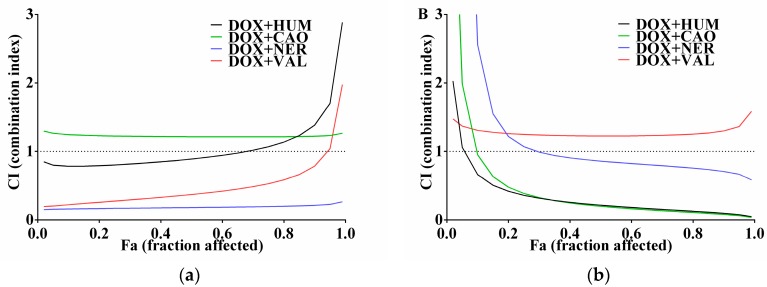

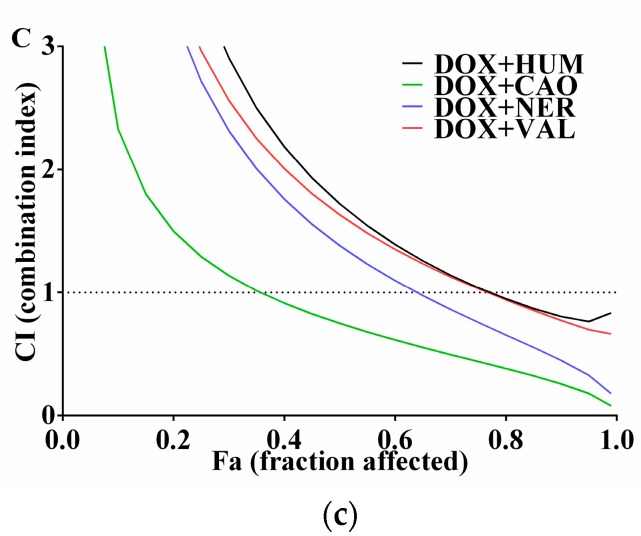
Combination indexes (CI) of DOX and sesquiterpenes in dependence of the fraction of affected cells (Fa) in ovarian cancer cell lines A2780 (**a**) and SKOV3 (**b**) and lymphoblast cell line CCRF/CEM (**c**). Incubations lasted for 72 h. Data were calculated using CalcuSyn software.

The use of drugs in combinations with natural compounds can cause significant shifts in efficacy in both positive and negative ways. The present study was aimed at testing and comparing the effect of selected sesquiterpenes on DOX efficacy in cancer cell lines with different sensitivity to DOX. The software CalcuSyn based on the Chou-Talalay method was used to evaluate the effect of DOX + sesquiterpene combinations [21]. Experiments were designed according to the recommendation in constant ratio. When both agents are cytotoxic as singles, the combination index (CI) calculation is required to study their combination effect. Graphs of CI versus fractions of dead cells (Fa) are presented in Figure 3. The resulting CI theorem of Chou-Talalay offers a quantitative definition for additive effect (CI = 1), synergism (CI < 1), and antagonism (CI > 1) in drug combinations. For cancer therapy, a Fa value > 0.8 (more than 80% affected cells) is more relevant than low Fa [21]. The values of CI in Fa = 0.8 for DOX + sesquiterpene combinations in cancer cell lines A2780, SKOV3 and CCRF/CEM are summarized in Table 2. As can be seen in Figure 3 and Table 2, the ability to affect DOX varied among sesquiterpenes as well as among cell lines. In the ovarian DOX-sensitive line A2780, NER showed a strong synergism with DOX. VAL acted also synergistically with DOX, but not in Fa > 0.9. CAO has a slightly antagonistic and HUM has an additive effect with DOX in this cell line. Ovarian cancer cell line SKOV3 is less sensitive to cytostatic drugs such as cisplatin and DOX [22]. However, combinations of DOX with HUM and CAO showed a strong synergistic effect. On the other hand, VAL had a mild antagonistic effect in SKOV3 cells. In lymphoblast cell line CCRF/CEM, a synergism of DOX + CAO and DOX + NER combinations were found. In CEM/ADR cells, DOX did not affect cell proliferation and for this reason, CalcuSyn could not be used. Nevertheless, these cells were treated with combinations of DOX (in constant concentration) with sesquiterpenes (in various concentrations), but these combinations had similar effects as did individual sesquiterpenes alone (data not shown).

### 2.3. Effect of Sesquiterpenes on Accumulation of DOX and Rhodamine 123 in Cancer Cells

**Figure 4:**
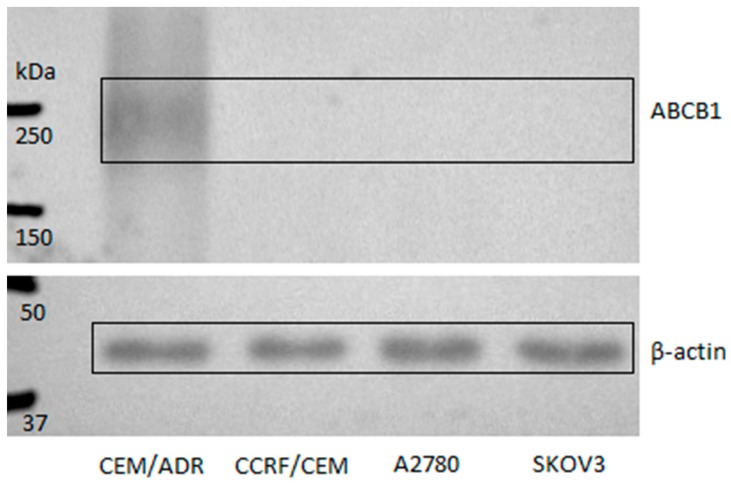
Immuno-quantification of ABCB1 level in the tested cancer cell lines. Protein β-actin was used as a reference housekeeping one.

The cancer cell lines used in our study significantly differed in sensitivity to DOX (see Table 1). Increased expression of the efflux transporter P-glycoprotein ABCB1 is often considered to be the mechanism of DOX resistance in cancer cells [23]. Therefore, we tested and compared ABCB1 protein level in all four cell lines using western blotting (see Figure 4). When antibodies with high specificity were used, a lot of ABCB1 protein was detected in CEM/ADR cells while no traces of this transporter were found in other cells. Previously, more efflux transporter proteins in cell line SKOV3 have been reported in comparison to cell line A2780 [24], but our results did not confirm that.

**Figure 5:**
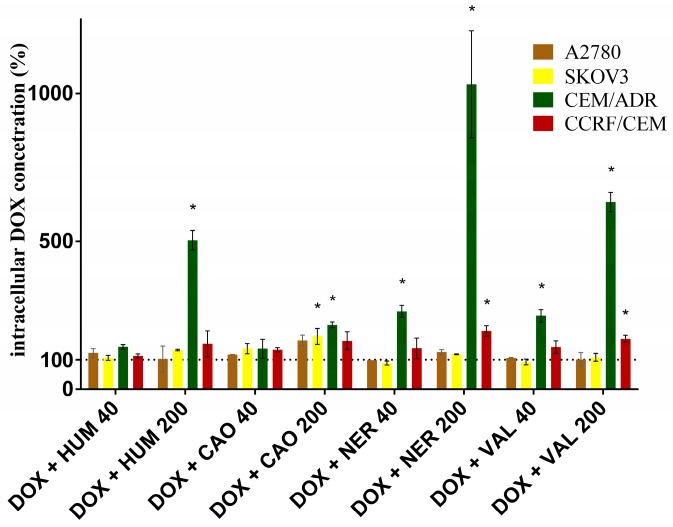
Effect of sesquiterpenes (40 and 200 µM) on intracellular DOX concentration. Data presented DOX intracellular concentration (in percentages), with DOX alone serving as control (100%). The colored bars represent the mean ± S.D. calculated from 3 independent measurements. The asterisk indicates a significant difference from the control cells (*p* < 0.05).

The accumulation of DOX within the cells after their treatment with DOX alone and with combinations of DOX + sesquiterpenes is compared in Figure 5. None sesquiterpene increased DOX accumulation in ovarian A2780 cells. In the ovarian cells SKOV3, only CAO in higher concentration was able to increase DOX accumulation. In lymphoblast CCRF/CEM cells, NER and VAL (only in higher concentrations) increased DOX accumulation 1.8 and 1.6 times, respectively. Other sesquiterpenes were ineffective. These results indicate that the increased DOX accumulation is not the main mechanism responsible for the synergistic action of sesquiterpenes with DOX in these cell lines. Other mechanisms e.g., the ability of sesquiterpenes to increase DOX-mediated formation of reactive oxygen species (ROS), which was observed in CaCo-2 cell line [11], could contribute to DOX-sesquiterpenes synergism. On the other hand, in the DOX-resistant lymphoblast CEM/ADR cells, all the tested sesquiterpenes were able to significantly increase intracellular DOX concentration, with NER being the most effective. In higher concentrations, HUM, CAO, NER and VAL increased DOX accumulation 5, 2, 10 and 6-times, respectively. In our previous study, the tested sesquiterpenes also increased intracellular DOX concentration in intestinal CaCo-2 cells, but the most effective was CAO. Moreover, the increase of DOX accumulation was less pronounced than in the lymphoblast CEM/ADR cells. None of the tested sesquiterpenes affected intracellular DOX concentration in the primary culture of rat hepatocytes [11].

**Figure 6:**
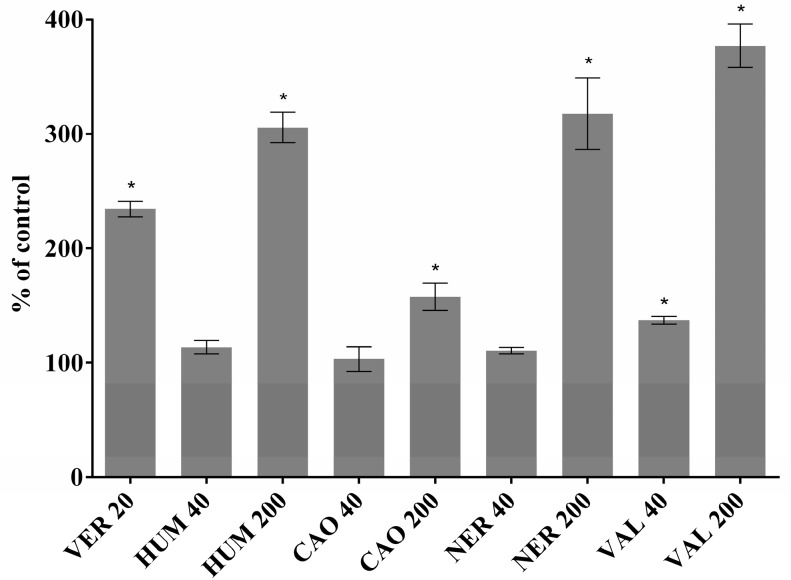
Fluorescence of RHO 123 in the CEM/ADR cell line. Untreated control cells represent 100%. Verapamil 20 µM was used as a positive control. Inhibition of efflux transporters is indicated by the increase of fluorescence. Data represent the mean ± S.D. calculated from 3 independent measurements (6 parallels in each). The asterisk indicates a significant difference from the control cells (*p* < 0.05).

Cell line CEM/ADR (as a DOX-resistant line with high level of ABCB1) was chosen to test the inhibitory effect of sesquiterpenes on Rhodamine 123 efflux. This substrate of the ABCB1 transporter is often used in activity assays in various tissues and cancer cells [25,26]. In our study, all the tested sesquiterpenes were able to inhibit the efflux of Rhodamine 123 (Figure 6). The ability of some derivatives of artemisin, another sesquiterpene, to inhibit ABCB1 activity in CCRF/CEM and CEM/ADR was previously reported [27].

## 3. Materials and Methods

### 3.1. Chemicals and Reagents

McCoy’s modified medium, RPMI-1640, *N*-2-hydroxyethylpiperazine-*N*′-2-ethanesulfonic acid (HEPES) buffer, sodium pyruvate, d-glutamine, resazurin, rhodamine 123 (RHO 123) and neutral red were supplied by Sigma-Aldrich (Prague, Czech Republic). Fetal bovine serum and gentamicin sulfate were purchased from Invitrogen (Carlsbad, CA, USA) and bovine serum albumin (BSA) from Fluka (Prague, Czech Republic). Doxorubicin and pure sesquiterpenes (α-humulene, β-caryophyllene oxide, trans-nerolidol, valencene) were supplied by Sigma-Aldrich. Stock solutions of sesquiterpenes were prepared in dimethyl sulfoxide (DMSO) and stored in the dark at 4 °C. All other chemicals used were of HPLC or analytical grade and provided by Sigma-Aldrich.

### 3.2. Cancer Cell Culture

Cell lines SKOV3 and A2780 were purchased from ATCC (supplier for Czech Republic: LGC Standards, Poland). Suspension cell lines CCRF/CEM and CEM/ADR5000 (CEM/ADR) were a gift from Prof. Wink, University of Heidelberg, DE. The cells were multiplied in three passages, frozen in aliquots and stored in liquid nitrogen. The absence of mycoplasma in all the cell lines used in the laboratory was periodically checked by Generi Biotech (Hradec Králové, Czech Republic). For every set of experiments (each lasting 3–9 weeks) new batch of stored cells was used. SKOV3 were cultured in McCoy’s modified medium with 10% FBS, A2780 in RPMI-1640 with 10% FBS and 2 mM d-glutamine, and CCRF/CEM and CEM/ADR were cultured in RPMI-1640 with 10% FBS, 1% HEPES, 1% sodium pyruvate. The resistance of CEM/ADR was maintained by incubation in 5 mM DOX for 24 h every week.

### 3.3. Tests of Cell Viability

The sesquiterpenes and DOX were pre-dissolved in DMSO or in pure ethanol. The cells were exposed to various concentrations of the tested compounds in the culture medium. The final concentration of organic solvent in medium was 0.1%. Cells cultured in medium with pure DMSO or ethanol without the tested compounds were used as control samples. 10% DMSO served as a positive control. The number of viable cells was assayed after 72 h using the neutral red uptake test for SKOV3 and A2780 or Alamar blue for the suspension cell lines.

### 3.4. The Neutral Red Uptake (NRU) Test

The cells were cultured in 96-well plates. After 72 h exposure, the medium was removed and 200 μL of neutral red-containing medium was added into each well, and the plates were incubated for an additional 3 h at 37 °C. The cells were washed with 100 μL of PBS, then fixed in a solution of 0.5% formaldehyde/1% calcium chloride for 15 min. The neutral red dye was released from the viable cells with a lysing solvent (50% ethanol/1% acetic acid) by shaking for 30 min at room temperature. The absorbance of solubilized dye was measured using a spectrophotometer (Infinite M200, Tecan, Zurich, Switzerland) at 540 nm. Each sample was assayed in 6 parallels and 3 independent experiments were performed. The viabilities of the treated cells were expressed as a percentage of untreated controls (100%). The effect of sesquiterpenes on DOX efficacy was evaluated using CalcuSyn software.

### 3.5. Alamar Blue (AB)

The cells were cultured in 96-well plates, followed by 72 h exposure with 10 µL of Alamar blue solution (0.1 mg resazurin/1 mL PBS). After 1 h incubation at 37 °C, fluorescence was measured (excitation 570 nm, emission 585 nm) using a fluoresce reader (Infinite M200, Tecan).

### 3.6. Accumulation of DOX in Cells

A suspension of SKOV3 and A2780 cells (600 μL; density 50,000 cells/mL per well) was set to a 12-well plate. After 48 h, 600 μL of solution in medium of the tested substances was added. The cells were treated with DOX (6 μM) alone, or in combination with individual sesquiterpenes (10 or 50 μg/mL) for 3 h. At the end of the experiment, the medium was removed and cells were washed with 500 μL of PBS. 100 μL of lysing buffer (25 mM Tris, 150 mM NaCl, 1% Triton, pH 7.4) added, at which time the cells were lysed for 15 min, then mixed with 300 μL of ice-cold methanol and centrifuged (10,000× *g*, 10 min, 4 °C). The supernatant was filtered through a 0.22 μm filter and the filtrate was used for analysis.

DOX was detected on an Agilent 1290 Series UHPLC chromatographic system (Agilent, Santa Clara, CA, USA) equipped with a Zorbax C18 Eclipse Plus (2.1 × 50 mm, 1.8 μm) column with a 1290 Infinity inline filter (Agilent). The HPLC method used by Skarka [28] was adapted to a UHPLC system: isocratic elution of 1.0 mL/min by 0.1% formic acid in water and acetonitrile in ratio 76:24; 30 °C thermostatic column compartment; fluorescence detection at λex = 480 nm and λem = 560 nm. Chemical standards were purchased from Toronto Research Chemicals (Toronto, ON, Canada).

### 3.7. Accumulation of RHO123

The suspension of CEM/ADR cells (106/mL) was incubated with RHO123 (2 h, on ice). After incubation the cells were washed by PBS and incubated with the tested compounds in serum free medium, with verapamil (20 µM) used as a control. After a 2 h incubation, the cells were washed by PBS and fluoresce measured by a flow cytometer (Accuri C6, Accuri Cytometers Europe Ltd., St. Ives, UK); excitation was undertaken with a by 488 nm argon laser, 530 nm emission filter. Flow cytometry data were analyzed by Flowing Software 2.

### 3.8. Western Blot Analysis

The cells were cultivated in 60 mm dishes, washed with PBS and lysed. Whole cell lysates were centrifuged (10 min, 13,000 rpm, 4 °C). Supernatant was normalized for protein by BCA assay. For separation, 7.5% acrylamide gel was used. Proteins were transferred to a PVDF membrane by wet blot (16 h, 20 V, 4 °C) and probed with anti-ABCB1 antibody (Cell Signaling No. 13342) and anti-ACTB antibody (Novus Biologicals, Abingdon, UK, NB600-501). Secondary antibody with horseradish peroxidase was used and the blots were imaged by chemiluminescence captured on film.

### 3.9. Statistical Analysis

The data are presented as means ± S.D. of a given number of experiments. Statistical significance (evaluated using a two-way ANOVA) was acceptable at a level of *p* < 0.05. The concentration inducing a 50% decrease of cell viability as compared to control (IC_50_) was calculated using GraphPad Prism 6.0 nonlinear regression.

## 4. Conclusions

In summary, drug interactions of sesquiterpenes with DOX were cell line specific. Some of the sesquiterpenes had a higher estimated IC_50_ than is normally achievable due to the lower solubility of the compounds. Only VAL had a measurable IC_50_ in all the tested cell lines, but in combination with DOX did not show a strong synergism in any cell line. Only NER showed a degree of synergism in all cell lines where it was possible to test for it. The effect from the combination of DOX with sesquiterpenes was not different from the effect of sesquiterpenes alone in the CEM/ADR cell line, despite the appearance of the largest shift of intracellular DOX concentrations. Further tests suggested the interaction of sesquiterpenes with efflux transporters as the possible mechanism of synergism with DOX.

## Figures and Tables

**Table 1:** IC_50_ values (µM) for sesquiterpenes and DOX after 72-h incubation—indicates the value was impossible to calculate. Calculated by CalcuSyn (version 1.1) software.

	A2780	SKOV3	CCRF/CEM	CEM/ADR
HUM	78	−	224	303
CAO	43	−	−	−
NER	119	−	120	232
VAL	61	114	203	302
DOX	0.16	1.41	0.037	−

**Table 2:** Overview of CI of DOX and sesquiterpenes in Fa = 0.8 calculated by CalcuSyn software.

	A2780	SKOV3	CCRF/CEM
HUM + DOX	1.1	0.13	0.95
CAO + DOX	1.2	0.11	0.38
NER + DOX	0.19	0.76	0.65
VAL + DOX	0.58	1.25	0.94
